# Prognostic and Immune Landscape Analysis of Ubiquitination-related Genes in Hepatocellular Carcinoma: Based on Bulk and Single-cell RNA Sequencing Data

**DOI:** 10.7150/jca.93425

**Published:** 2024-03-11

**Authors:** Zibo Yuan, Qingwei Zhu, Qingsong Wu, Zhe Zhang, Junwei Guo, Gongqiang Wu, Cuiping Zheng, Qiuran Xu, Dongsheng Huang, Di Cui

**Affiliations:** 1 Qingdao Medical College, Qingdao University, Qingdao, 266000, China.; 2 The Key Laboratory of Tumor Molecular Diagnosis and Individualized Medicine of Zhejiang Province, Zhejiang Provincial People's Hospital (Affiliated People's Hospital), Hangzhou Medical College, Hangzhou, 310000, China.; 3 The Second Clinical Medical College of Zhejiang Chinese Medical University, Hangzhou, 310000, China.; 4 Department of Hematology, Dongyang People's Hospital of Zhejiang Provincial, Dongyang, 322100, China.; 5 Department of Hematology and Chemotherapy, Wenzhou Central Hospital, Wenzhou, 325000, China.; 6 General Surgery, Cancer Center, Department of Hepatobiliary and Pancreatic Surgery and Minimally Invasive Surgery, Zhejiang Provincial People's Hospital (Affiliated People's Hospital), Hangzhou Medical College, Hangzhou, 310014, China.

**Keywords:** hepatocellular carcinoma, ubiquitination, tumor immune microenvironment, immune checkpoints, risk model, drug sensitivity

## Abstract

**Background:** Despite significant advances in tumor immunotherapy, hepatocellular carcinoma (HCC) remains a malignancy with a challenging prognosis. The increasing research emphasizes the crucial role of ubiquitination in tumor immunotherapy. However, the establishment of prognostic signatures based on ubiquitination-related genes (UbRGs) and their role in immunotherapy are still lacking in HCC.

**Methods:** We employed datasets from TCGA and GEO for transcriptome differential expression analysis and single-cell RNA sequencing analysis. Applying weighted gene co-expression network analysis, cox regression, lasso, selection and visualization of the most relevant features, and gradient boosting machine, we identified hub UbRGs as a gene signature to develop a prognostic model. We evaluated the predictive utility concerning clinical characteristics as well as its role in the immune landscape and immunotherapy potential. Additionally, western blotting, reverse transcription-quantitative PCR, and immunofluorescence were employed to detect the expression and sub-localization of hub genes.

**Results:** Three hub UbRGs (BOP1, CDC20, and UBE2S) were identified as a gene signature. In particular, the high-risk group exhibited notable characteristics, including higher tumor mutation burden, enrichment in immune-related pathways, up-regulation immune checkpoint, and higher immunity scores. Treatment response to immunotherapy varied based on the expression of PD-1 and CTLA-4. Furthermore, single-cell data analysis revealed heterogeneous expression of hub UbRGs across different cell subtypes, while cytological experiments provided additional confirmation of the high expression of hub UbRGs in HCC.

**Conclusion:** Our study provides valuable insights into the identification of novel ubiquitination-related biomarkers with potential applications for prognosis, immunotherapy prediction, and drug sensitivity in HCC.

## Introduction

The liver is ranked as the sixth most common primary site for cancer and hepatocellular carcinoma (HCC) is the third leading cause of global cancer-related mortality 1. Notably, the immune system exerts significant influence over the progression of HCC. The immune microenvironment plays a critical role in the development and advancement, and distinct immune characteristics associated with its various etiologies have been identified in HCC 2, 3. As the initial treatment, systemic therapies, typically sorafenib or lenvatinib, are administered to approximately 50% of HCC patients 4. Inhibitors targeting immune checkpoints have revolutionized cancer treatment and gained significant attention 5, 6. Vaccination, virotherapy, and other immune system-targeting approaches are also under development. Despite these remarkable breakthroughs, patients with HCC still face poor survival prospects and lack additional biomarkers to aid in therapeutic decision-making 7. Therefore, it is essential to identify accurate diagnostic, therapeutic, and prognostic markers.

Ubiquitination is a multistep process involving E1 enzyme activation, E2 conjugation, and E3 ubiquitin ligation to the substrate protein. Depending on the type of polyubiquitin chain formed, ubiquitination can direct the substrate to multiprotein complexes or target it for degradation via proteasomes 8. This process is dynamic and reversible relying on deubiquitinases to remove ubiquitin and stabilize substrate proteins 9. In the context of cancer development, ubiquitination plays a pivotal role, such as angiogenesis, apoptosis, metastasis, and immunity through its diverse effects on transcription, post-translation modifications, and protein levels 8. Pathological ubiquitination has been associated with promoting cancer development and evasion of the immune system 10. Recent research has demonstrated that ubiquitination plays essential roles in pathways related to carcinoma, and the accumulation of ubiquitinated proteins may offer a novel approach for cancer treatment 11. USP22 can regulate PD-L1 by deubiquitination and modulate the infiltration of T cells into malignancies 10. To prevent immune escape, RNF125 enhances the ubiquitination and degradation of PD-L1 12. These findings suggest the importance of ubiquitination in tumor immunity. However, they do not elucidate the specific mechanisms by which it regulates immune cells and their microenvironment. Therefore, further studies are needed to explore the role of ubiquitination in regulating immune responses in HCC.

In this study, we utilized the TCGA-LIHC, GSE76427, and GSE149614 datasets to identify hub genes of ubiquitination-related genes (UbRGs) by analyzing bulk and single-cell RNA sequencing data (scRNA-seq). Additionally, we validated an HCC diagnostic model. Furthermore, we investigated the interrelationship among gene expression, survival, tumor immune microenvironment (TME), mutation analyses, and hub gene expression. Hub genes were validated using western blot (WB), real time quantitative PCR (RT-qPCR), immunofluorescence (IF), and the HPA database. The comprehensive understanding of the multimolecular characteristics of UbRGs is expected to contribute to the development of a distinctive and promising approach for identifying HCC biomarkers and supporting future research endeavors.

## Materials and Methods

### Acquiring and Processing Transcriptome Data

**Figure 1:**
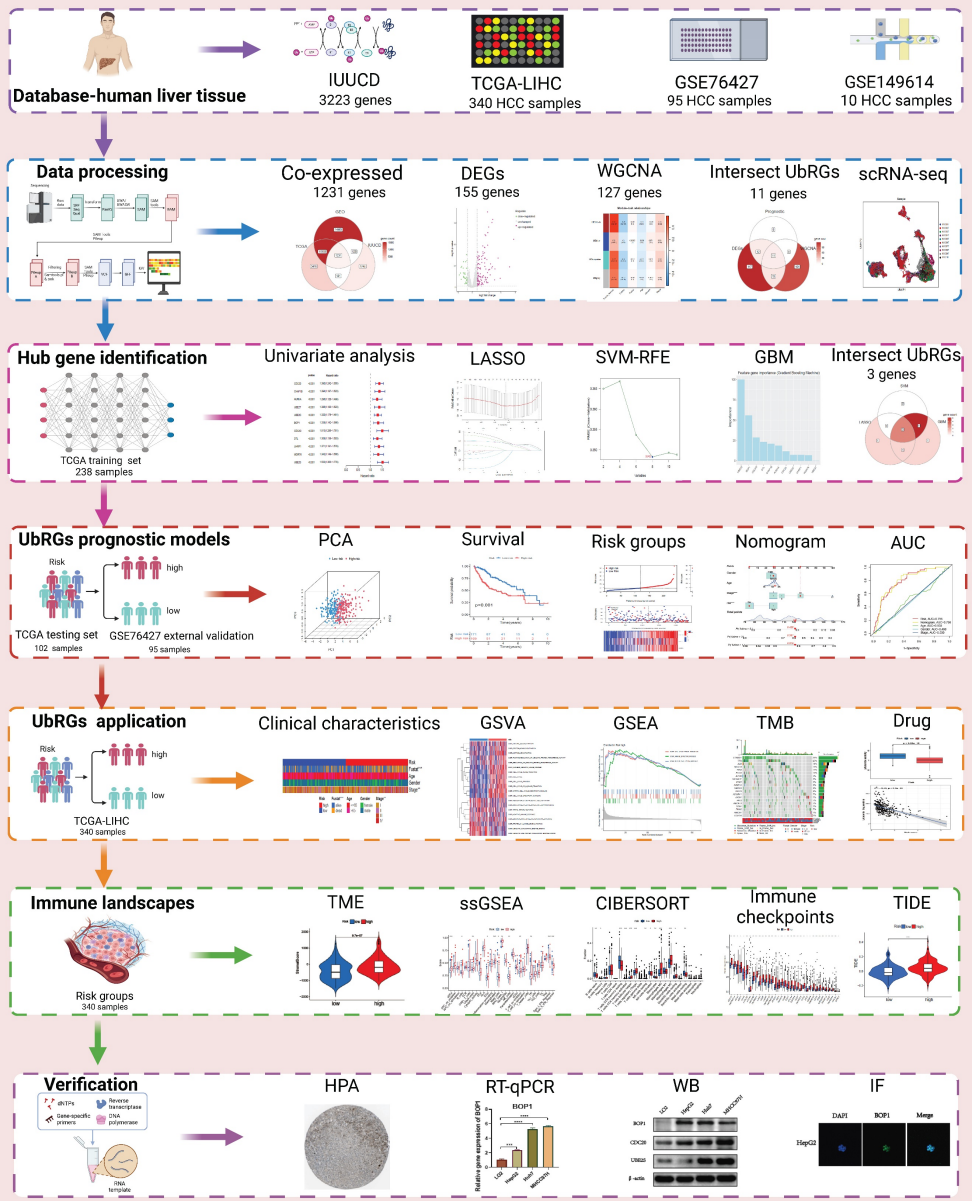
** Flowchart of this study.** The workflow of the analysis steps (Created with BioRender.com).

The TCGA database contains expression profiles, genetic mutations, and clinical information for LIHC (N=369). Patients without clinicopathological records or with overall survival (OS) times less than 1 month were excluded from this study. A total of 340 patients were enrolled and divided into a training group and a testing group in a 7:3 ratio. The model was constructed using the training group, and its reliability and efficiency were evaluated using the testing group. For subsequent analysis, a log2 transformation was applied to all TPM data. The GEO database provided matrix files, essential clinical information, and survival data for the independent HCC dataset GSE76427 (N = 115). Patients who had a survival of less than one month were excluded. Ultimately, 95 patients were included in the study for external validation (Table S1). The comprehensive workflow of the study is illustrated in Figure 1.

### Screening of UbRGs

The iUUCD 2.0 database provided information on UbRGs 13. A total of 3223 genes were retrieved for subsequent studies (Table S2). The mRNA expression profiles of 1231 UbRGs were extracted from the GSE76427 and TCGA databases for further analysis. WGCNA has been effectively utilized in numerous biological studies 14-16. The "WGCNA" package was used to transform the similarity matrix into an adjacency matrix using a weight coefficient of 15. Combining highly similar modules resulted in a coexpression network. Among these modules, the brown module showed the highest correlation and was considered to have the strongest association with tumors (Table S3). Among the UbRGs, we found 155 different expressed genes (DEGs) based on FDR < 0.05 and |log2FC| > 1. Among the 155 UbRGs, 23 genes with expression variations were associated with a negative prognosis. The brown module, DEGs, and prognoses identified 11 UbRG genes.

### Construction of UbRGs Signature

Eleven UbRG genes were identified based on the brown module, DEGs, and prognosis, and we identified prognostic UbRGs using univariate cox regression. Subsequently, the least absolute shrinkage and selection operator (LASSO), support vector machines-recursive feature reduction (SVM-RFE), and gradient boosting machine (GBM) machine learning algorithms were employed for screening characteristic genes. LASSO, a dimensionality reduction technique, evaluates high-dimensional data more effectively compared to regression analysis. We used the “glmnet” package to perform LASSO analysis with a tuning/penalty parameter and 10-fold cross-validation. Significant genes were selected through GBM analysis. SVM-RFE outperforms linear discriminant analysis and mean squared error in detecting important and redundant attributes. The genes screened by the machine learning algorithms are listed in Table S4.

### Construction and Validation of Prognostic Models

Significant key genes were identified through machine learning analysis and integrated into a multivariate cox regression model (Table S5). Prognostic signatures based on UbRGs were established using multivariate cox proportional in the training cohort. Additionally, for each LIHC sample, the risk score was calculated based on the following formula:



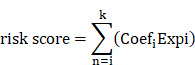



Based on the median risk score, all cohorts in the training set, testing set, and external validating set were classified into low and high risk groups. Subsequently, we analyzed the performance of this prognostic index in all cohorts using survival analysis and receiver operating characteristic (ROC) curve. Additionally, univariate and multivariate prognostic models were employed to assess the reliability of this ubiquitination-based prognostic index as an indicator. In order to determine the probabilities of OS at 1, 3, and 5 years, a nomogram was developed incorporating the risk score and clinical features as prognostic factors.

### Tumor Mutation Burden (TMB)

We utilized the "maftools" package to analyze and visualize mutation data using LIHC data. Non-synonymous and frameshift indels were considered, with a detection limit of less than 5%. The patients were classified into four groups based on their mean TMB and risk classification.

### Analyses of Pathway and Functional Enrichment

The "clusterProfiler" package was employed to perform KEGG and GO enrichment analysis of biological processes and pathways. In addition, we employed the "GSVA" package to investigate differences in the levels of biological process activities among the TCGA samples.

### Immune Landscapes Related to the UbRGs Signature

The "ESTIMATE" package was used to predict TME scores for LIHC. To assess immunocyte infiltration in the TME, we employed CIBERSORT and ssGSEA to estimate the density of immune cell infiltration in patients. The box plot diagram illustrated the differences between the two subtypes.

### Immune Checkpoint and Immunotherapy Assessments

The expression of immune checkpoint genes was correlated with the response to immunotherapy. Moreover, Spearman correlation analysis identified the hub UbRGs that were highly correlated with immune checkpoints. The tumor immune dysfunction and exclusion (TIDE) score table was obtained from the TIDE database using transcriptome files as the data source. To validate the immune checkpoint blockade treatment cohort, we selected the IMvigor210 dataset. Using the IMvigor210 program, we extracted gene expression data and assessed the immunotherapeutic efficacy in a publicly available cohort from a previous study 17, and a violin plot was created to visualize the results.

### Drug Sensitivity Analysis of Risk Groups

We utilized the "oncoPredict" package to predict the half maximal inhibitory concentration (IC50) levels of HCC samples in LIHC for different antineoplastic drugs. The IC50 values and risk score were then subjected to a Spearman correlation analysis in order to identify sensitive and resistant drugs (|R| > 0.3 and *p* <0.05).

### Processing and Acquiring scRNA-seq Data

The GSE149614 dataset comprises ten HCC samples in total. The quality of the scRNA-seq data was analyzed using the "Seurat" and "SingleR" packages. To ensure high-quality scRNA-seq data, we selected cells with less than 20% mitochondrial genes and genes showing expression levels between 200 and 7,000. A total of 34,061 cells were considered eligible for further study. Following data normalization, we employed the "FindVariableFeatures" function to identify the top 2,000 hypervariable genes. Due to the data being derived from multiple samples, we employed the harmony method to remove batch effects that could confound subsequent analysis. We utilized the "IntegrateData" and "ScaleData" functions to achieve proper data integration and scaling. The top 20 principal components were analyzed to identify meaningful clusters. Cell cycle markers included in the "Seurat" packages were utilized to assess the heterogeneity of the cell cycle. Additionally, we selected cell marker genes using the marker genes from the CellMarker 2.0 database (Table S6) 18. Cell-type copy number variation (CNV) was predicted using the "infercnv" package to distinguish cancer cells from normal cell types. Single-cell data processing is illustrated in Figure S1. The "AUCell" package was employed to determine the enrichment scores of hub UbRGs for cell subtypes in the scRNA-seq dataset. Subsequently, each cell's UbRGs score was calculated based on the three hub genes using the “AddModuleScore” function.

### Reverse Transcription-quantitative (RT-qPCR)

Human liver cancer cell lines (HepG2, Huh7, and MHCC97H) were purchased from ServiceBio, and human normal liver cell lines (LO2) were purchased from iCell Bioscience. RNA extraction was performed using an RNA rapid purification kit (ES science). The concentration and purity of RNA were assessed using Nano-Drop One (Thermo Fisher Scientific) by measuring the absorption ratio at 260-280 nm, which should fall within the range of 1.9 to 2.2. Subsequently, cDNA synthesis was performed using the s1000 instrument (Bio-Rad Laboratories) and the PrimeScript RT reagent kit (Takara Bio). RT-qPCR was conducted with SYBR Green (Shanghai Yeasen Biotechnology Co) following the manufacturer's protocol. The primer sequences for hub UbRGs and β-Actin were shown in Table S7.

### Western Blotting (WB)

The total protein content of cells was extracted using lysis buffer (Beyotime Institute of Biotechnology), phenylmethylsulfonyl fluoride solution (Beyotime Institute of Biotechnology), and a protease inhibitor cocktail (bimake). The protein concentration was determined using the Pierce BCA protein assay kit (Thermo Fisher Scientific). The 0.45 µm membrane was blocked with 5% skim milk. The membrane was incubated with anti-UBE2S (Proteintech), anti-BOP1 (Proteintech), anti-CDC20 (Proteintech), and anti-β-ACTIN (Affinity Biosciences). The membranes were washed three times and incubated with a secondary antibody (Thermo Fisher Scientific).

### Immunofluorescence (IF)

Cells growing on coverslips were fixed for ten minutes at room temperature using a 3.7% formaldehyde solution. The cells were permeabilized with PBS containing 0.1% Triton X-100 (Beyotime) and then blocked with PBS containing 5% BSA (Beyotime). The cells were incubated overnight at 4°C with antibodies diluted in PBS containing 3% BSA, followed by 1 hour of detection with Alexa Fluor 488-conjugated secondary antibody (Beyotime). Nuclei were identified by DAPI (Beyotime) for 3 minutes.

### Statistical Analysis

Statistical analyses were performed using R 4.2.2. The experiment's results were statistically analyzed using GraphPad Prism version 8.0. Continuous variables were described using mean and standard deviation. For continuous variables with a normal distribution, Student's t-tests were used; for non-normal variables, Wilcoxon rank-sum tests were used; and for categorical variables, Chi-square or Fisher's exact tests were used. Gene correlations were determined using Pearson correlation analysis. Statistical significance was defined as *p* < 0.05 (two-tailed).

## Results

### Identification of UbRGs Signature through WGCNA and Machine Learning

**Figure 2:**
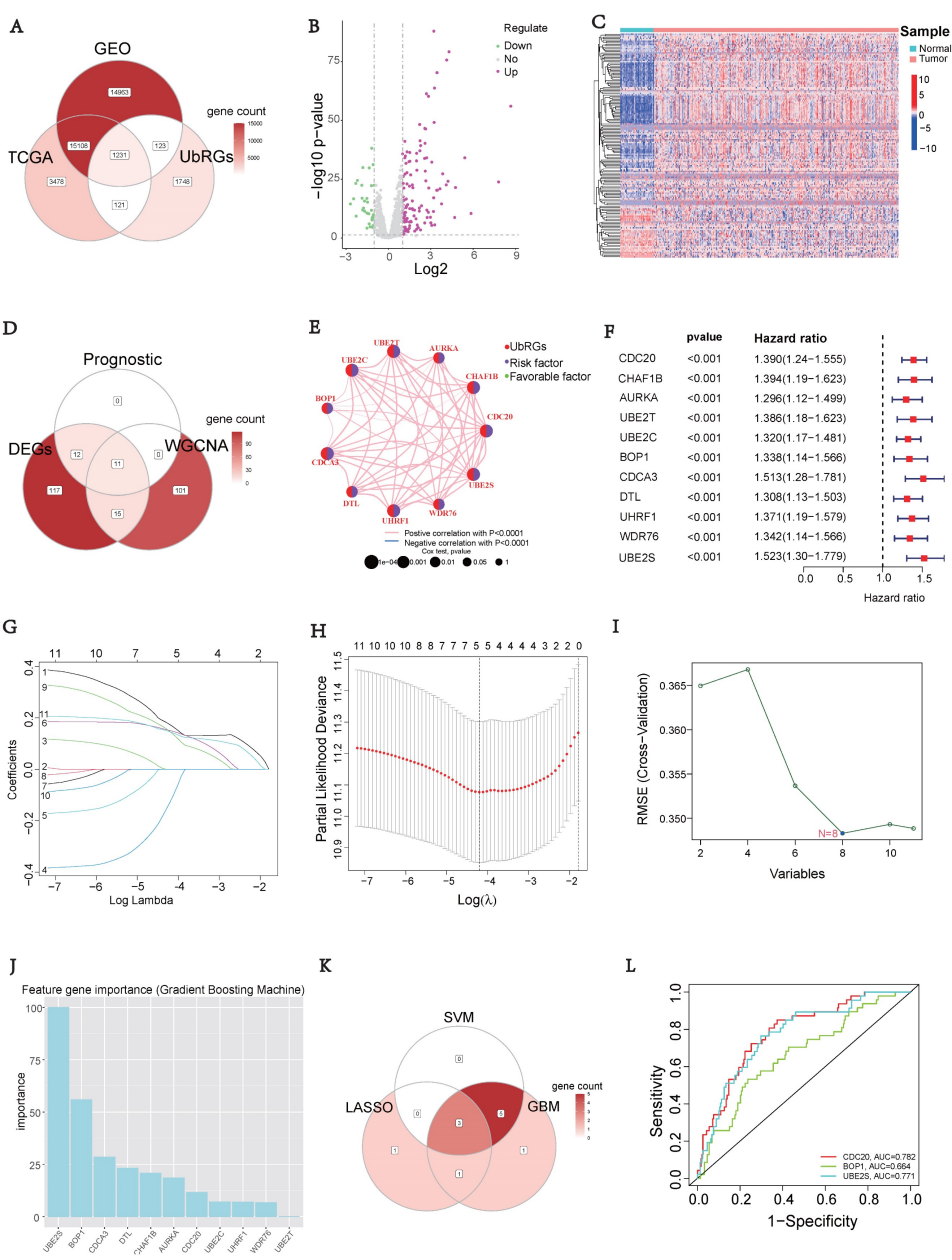
** Signature of Ubiquitination-Related Genes (UbRGs).** (A) The Venn diagram illustrates the overlap of co-expressed genes among the TCGA, GEO, and IUUCD 2.0 databases. (B) The volcano plot visualizes the differentially expressed genes (DEGs) between tumor and normal tissue among the 1231 UbRGs. (C) The heatmap displays the expression pattern of 155 characteristic DEGs. (D) The Venn diagram illustrates the overlap of UbRGs among WGCNA, DEGs, and prognosis. (E) The network diagram visualizes the interactions among eleven genes. (F) The forest plot displays the expression of the eleven OS-related genes. (G) The distribution of coefficients in the LASSO model. (H) Selection of the five optimal parameters in the LASSO model. (I) Validation of the expression of biomarker signature genes using the SVM-RFE algorithm. (J) Importance of feature genes are determined by the gradient boosting machine (GBM) algorithm. (K) The Venn diagram illustrates the overlap of diagnostic markers extracted by the LASSO, SVM-RFE, and GBM algorithms. (L) ROC curves are plotted for CDC20, BOP1, and UBE2S.

To investigate the prognostic value of UbRGs in LIHC patients, a Venn diagram was used to visualize the co-expression of 1231 genes across three independent datasets: TCGA, GEO, and IUUCD (Figure 2A). The volcano plot was generated to illustrate the expression patterns of 1231 co-expressed genes in normal and tumor tissues (Figure 2B). Differential analysis of these co-expressed UbRGs between tumor and normal groups revealed 155 genes with significant differential expression. The heatmap showed that the majority of DEGs exhibited higher expression in tumor tissues (Figure 2C).

In addition, we conducted a WGCNA using 1231 UbRGs from the intersection to identify candidate genes significantly associated with clinical features (Figure S2). Anomalous samples were excluded by clustering the sample dendrogram and trait indicator based on Pearson correlation coefficients (Figure S2A). Patients with comprehensive information including age, gender, sample, futime, fustate, and stage were included in the study. The soft threshold of 15, which corresponded to the lowest mean connectivity, was selected (Figure S2B). The MEDissThres was set to 0.08, resulting in the formation of four modules (Figure S2C). The blue module comprised 214 genes, the brown module comprised 127 genes, and the turquoise module comprised 422 genes. The grey module consists of 466 genes that did not belong to any other module (Figure S2D). Correlation analysis showed a significant correlation between the brown module and the clinical features of LIHC (Figure S2E). Indeed, the membership of the brown module genes exhibited a significant correlation with clinical characteristics (Figure S2F). The connectivity between the 127 genes identified as important genes in the brown module was calculated for further analysis.

Furthermore, we intersected the differentially expressed UbRGs associated with prognosis and genes selected through WGCNA, resulting in the identification of 11 key genes for survival analysis: CDC20, CHAF1B, AURKA, UBE2T, UBE2C, BOP1, CDCA3, DTL, UHRF1, WDR76, and UBE2S (Figure 2D). Figure 2E illustrated the positive correlations among these 11 UbRGs. Forest plot analysis confirmed these 11 UbRGs as risk factors for HCC patients (Figure 2F). To further select UbRGs, three machine learning algorithms (LASSO, GBM, and SVM-RFE) were employed, which highlighted three hub UbRGs (CDC20, BOP1, and UBE2S) for constructing a risk model (Figures 2G-K). Finally, the predictive performance of those hub genes was excellent when evaluated using time-dependent ROC curves (Figure 2L).

### Construction of UbRGs-related Riskscore Model

**Figure 3:**
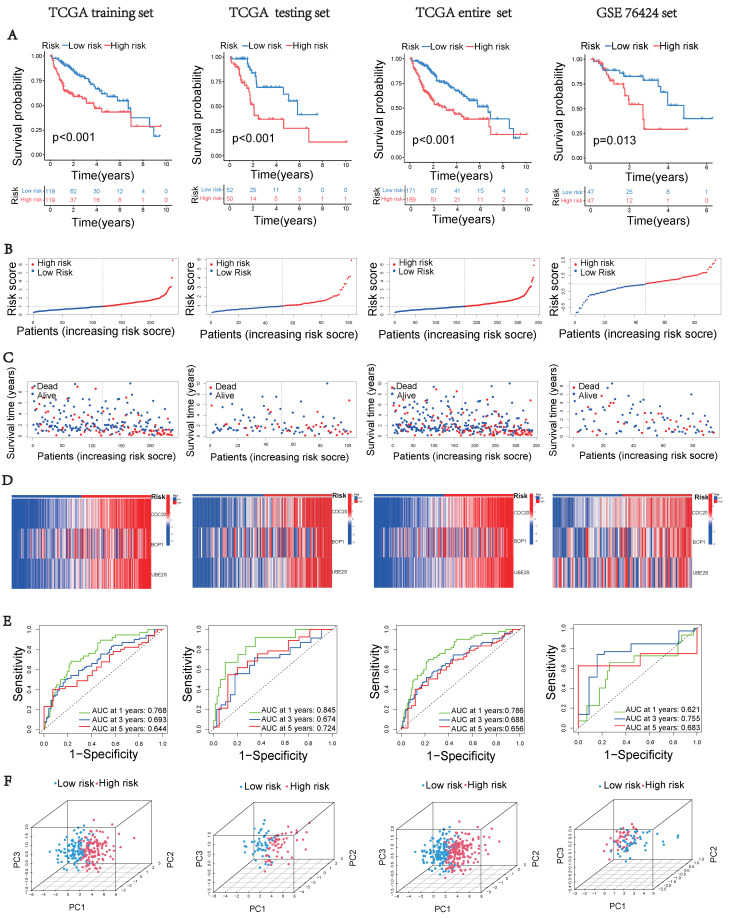
** Construction of the UbRGs risk score model.** (A) Kaplan-Meier curves for OS based on the stratification of risk scores in different datasets. (B) Distribution of risk scores in different datasets. (C) Distribution of survival time of patients in different datasets. (D) Heatmap illustrating the expression levels of CDC20, BOP1, and UBE2S in different datasets. (E) ROC analysis for the prediction of OS at 1, 3, and 5 years in different datasets. (F) Principal component analysis of different datasets.

Based on the expression of hub UbRGs (CDC20, BOP1, and UBE2S), the risk score of HCC patients was calculated using a multivariate Cox proportional hazards model. The median risk score was used to categorize HCC patients in the training and validation sets into low-risk and high-risk groups. For internal validation, the test set and the entire dataset were used, while for external validation, the independent dataset GSE76427 was employed. The survival curves indicated significantly lower survival probability in high-risk patients compared to low-risk patients in both internal and external test sets (*p* < 0.05) (Figure 3A). The mortality rate increased with higher risk scores based on the risk score and outcome from different sets (Figures 3B-C). Moreover, the heatmap showed higher expression levels of three hub UbRGs in high-risk patients, which was consistent with the findings of the external validation set (Figure 3D). To further evaluate the predictive accuracy of three hub genes for the prognosis of HCC patients, ROC curve analysis was performed on the training set and various test sets, resulting in AUC values all greater than 0.6 (Figure 3E). The model successfully distinguished HCC patients through PCA analysis of different sets (Figure 3F). These findings suggest that the prognostic model associated with ubiquitination hub genes can effectively predict the survival rate of HCC.

### Prognostic Model Clinical Validation

**Figure 4:**
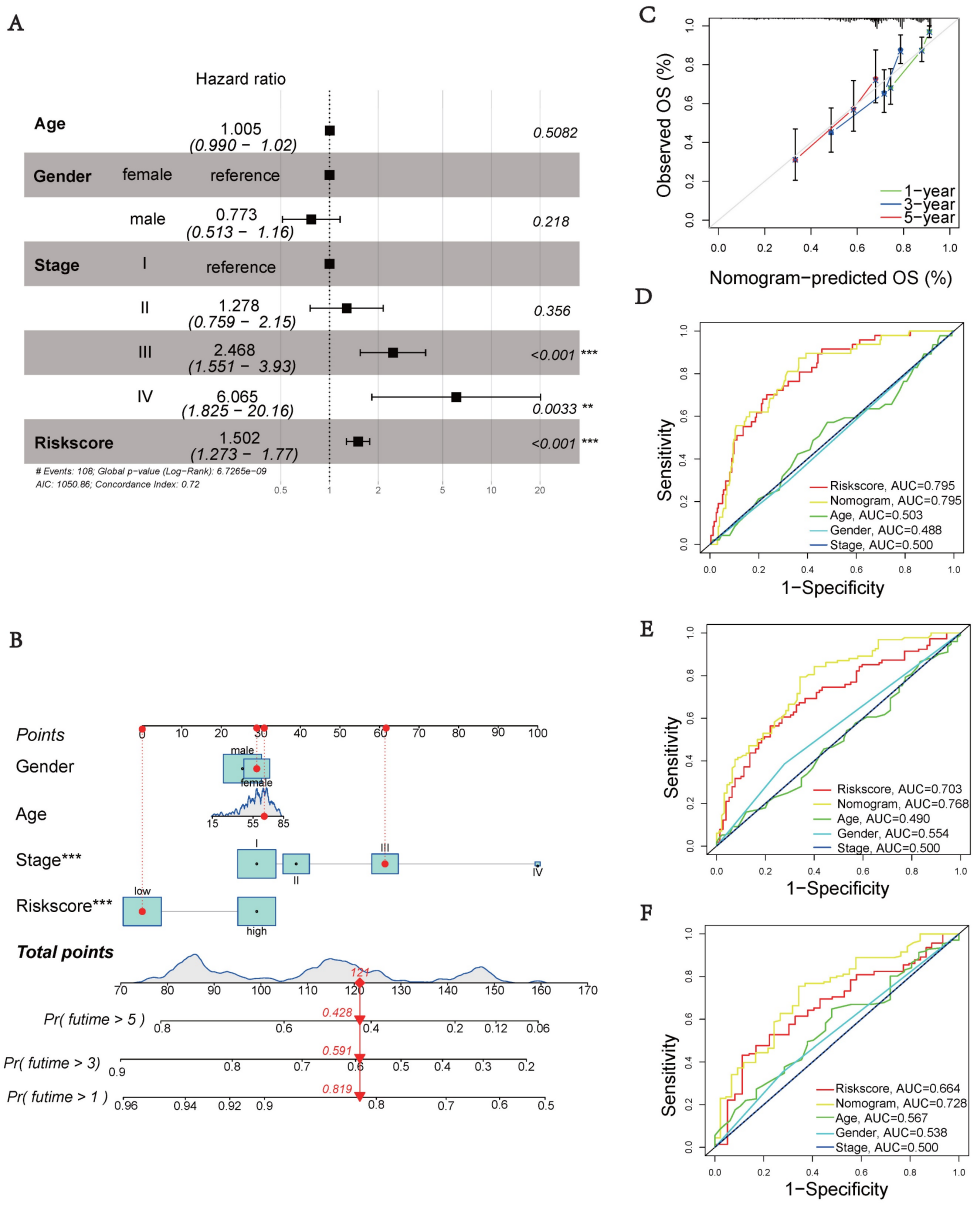
** Clinical validation of the prognostic model.** (A) Analysis of risk score and clinicopathological features using a forest plot. P-values were calculated using Mann-Whitney U tests. (B) Nomogram for predicting OS. (C) Calibration plot of the nomogram comparing projected and observed outcomes. (D) AUC values for predicting 1-year OS. (E) AUC values for predicting 3-year OS. (F) AUC values for predicting 5-year OS.

Next, we performed multivariate cox regression analysis using clinical information and risk scores to identify independent prognostic factors. The analysis revealed that stage III (HR = 2.468, 95% CI: 1.551-3.930, *p* < 0.001), stage IV (HR = 6.065, 95% CI: 1.825-20.16, *p* = 0.0033), and riskscore (HR = 1.502, 95% CI: 1.273-1.770, *p* < 0.001) were identified as independent prognostic factors (Figure 4A). To accurately and conveniently quantify HCC, we developed a nomogram that incorporates age, gender, stage, and riskscore to predict OS. By utilizing this nomogram, we can provide a more accurate assessment of patient risk and make informed decisions regarding future treatment strategies (Figure 4B). To evaluate the prediction accuracy of the nomogram, we employed a calibration curve that compares the observed outcomes with predicted outcomes (Figure 4C). The AUC values at 1, 3, and 5 years were 0.795, 0.768, and 0.728, respectively (Figures 4D-F). These values were significantly higher than the AUC values associated with clinical characteristics. These results suggest that the riskscore serves as an independent prognostic indicator, and the nomogram accurately predicts the prognosis of HCC patients.

### Tumor Mutation Characteristics

**Figure 5:**
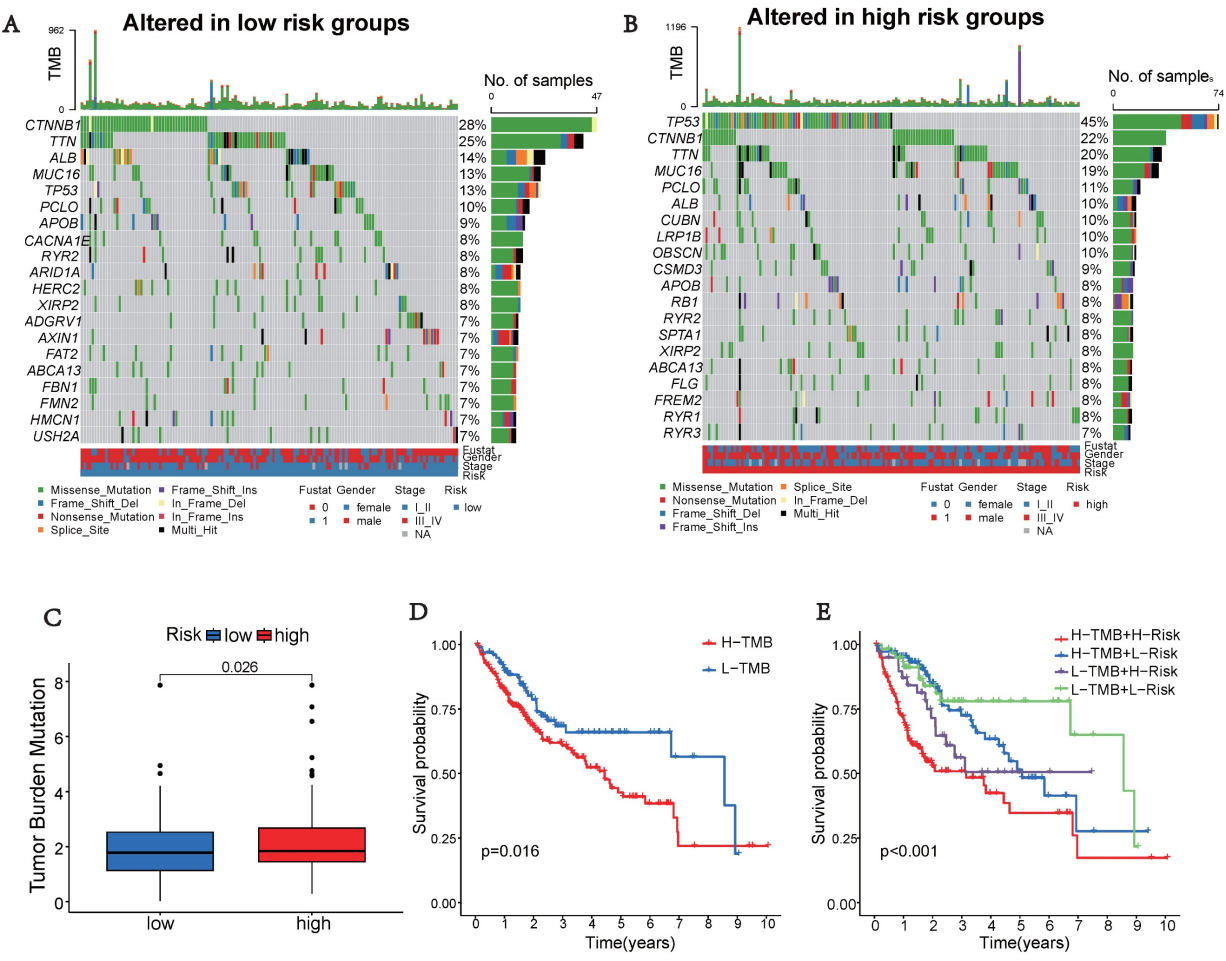
** Tumor mutation characteristics in different subgroups based on UbRGs.** (A) Mutation type and number in low risk groups. (B) Mutation type and number in high risk groups. (C) Differences in Tumor mutation burden (TMB) between the two risk groups. (D) Kaplan-Meier survival analysis of TMB. (E) Effects of combined UbRGs risk score and TMB.

Somatic mutations were also evaluated in the distinct risk groups. Figures 5A-B displays the top 20 genes with the highest mutation frequencies in each risk category. In the low-risk group, the five most frequently altered genes were CTNNB1 (28%), TTN (25%), ALB (14%), MUC16 (13%), and TP53 (13%), while the high-risk group was characterized by TP53 (45%), CTNNB1 (22%), TTN (20%), MUC16 (19%), and PCLO (11%) as the top five most mutated genes. There was a significant difference in TMB, with the high-risk group showing a higher frequency of tumor mutations compared to the low-risk group (*p* = 0.026) (Figure 5C). Additionally, patients showed a significantly improved prognosis (*p* = 0.016) in the low-risk group (Figure 5D). Moreover, our prognostic analysis, integrating TMB and UbRGs, demonstrated that patients with low risk and low TMB had the most favorable prognosis, while patients with high risk and TMB had the poorest prognosis (Figure 5E).

### Gene Function Enrichment Analysis

**Figure 6:**
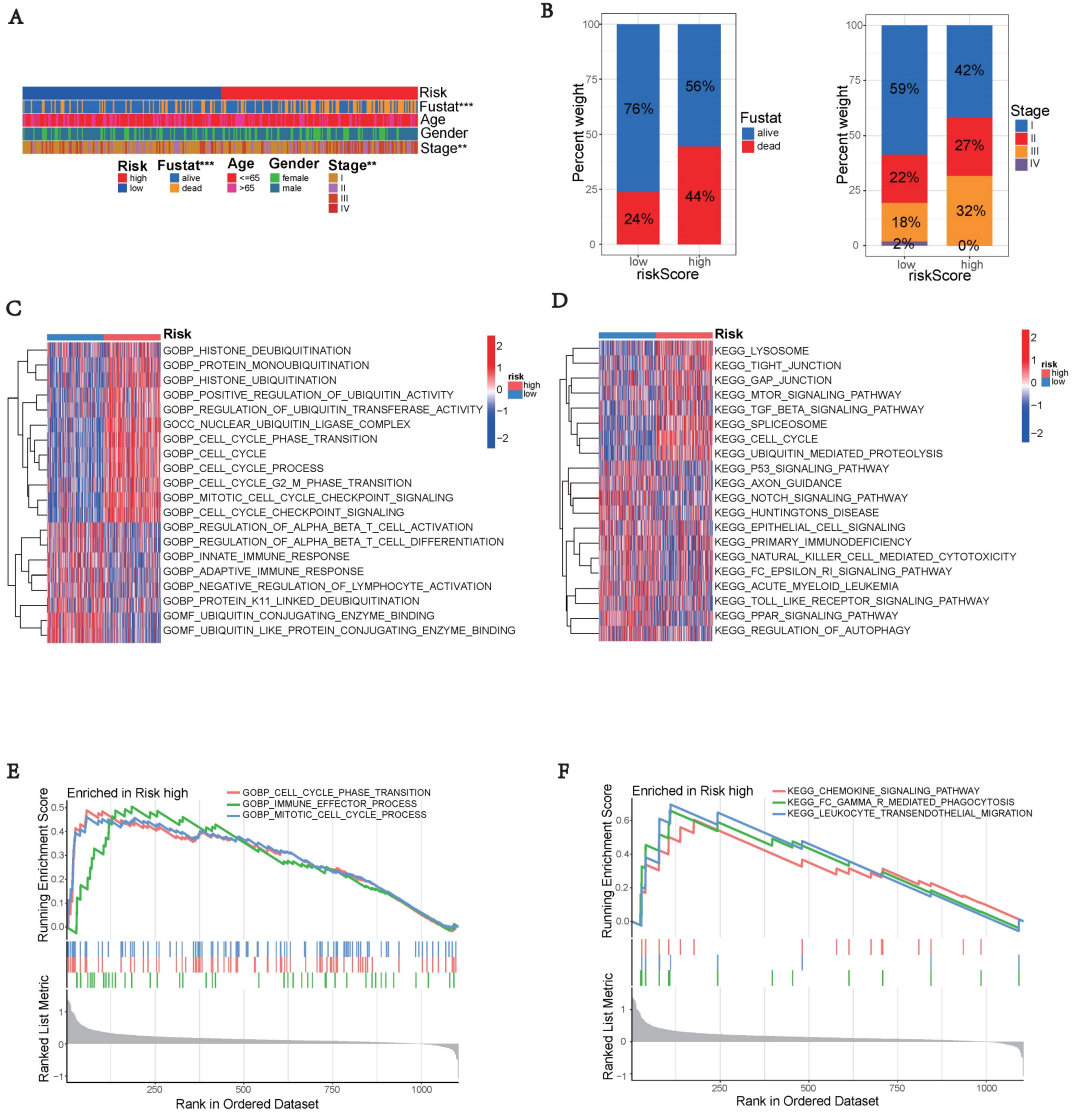
** Enrichment analysis of gene functions in risk groups using gene set variation analysis (GSVA) and gene set enrichment analysis (GSEA).** (A) Heatmap illustrating the clinical characteristics of patients in the low-risk and high-risk groups. (B) Bar graphs depicting the distribution of different risk groups based on patient survival status and tumor stage. (C) Heatmap illustrating the activation state of GO pathways in different groups after processing using GSVA. (D) Heatmap illustrating the activation state of KEGG pathways in clustered groups after processing using GSVA. (E) Enriched GO pathways associated with DEGs in the predicted groups using GSEA. (F) Enriched KEGG pathways associated with DEGs in the predicted groups using GSEA.

The high-risk group exhibited significantly worse survival and tumor pathology compared to the low-risk group (Figure 6A). Additionally, the high-risk group had a higher proportion of patients who were Stage II and III compared to the low-risk group (Figure 6B).

To explore the potential functional differences between the two risk groups defined by (three) hub UbRGs, we performed differential expression analysis and functional annotation. GO enrichment analysis revealed the involvement of DEGs in various biological processes, such as mitotic cell cycle, checkpoint signaling, cell cycle, histone ubiquitination and deubiquitination, regulation of T cell activation, innate immune response, adaptive immune response, negative regulation of lymphocyte activation, and other processes (Figure 6C). KEGG pathway analysis identified alterations in multiple signaling pathways, including lysosomes, mTOR signaling pathway, spliceosome, p53 signaling pathway, and NK cell-mediated cytotoxicity (Figure 6D). Moreover, we investigated the disparities in biological functions between the two risk groups using GSEA. The analysis results revealed that the high-risk group showed enrichment of diverse biological processes, including cell cycle phase transition, immune effector process, and mitotic cell cycle process (Figure 6E). Additionally, pathways such as FC-mediated phagocytosis, chemokine signaling pathway, and leukocyte transendothelial migration were also enriched. The enrichment analysis results revealed a complex relationship between hub UbRGs and biological processes pathways of the immune system (Figure 6F). These findings unveiled distinct pathophysiological mechanisms underlying HCC and suggested that this complexity might contribute to the differences in disease progression and patient outcomes.

### Landscape of Immunity

The tumor microenvironment, especially tumor-infiltrating immune cells, plays a crucial role in tumor progression and therapeutic outcomes 19. The pathway analysis above indicated that the high-risk group, determined by the ubiquitination score, exhibited enrichment in immune system-related pathways. Therefore, we investigated the variations in the TME and immune cell infiltration.

**Figure 7:**
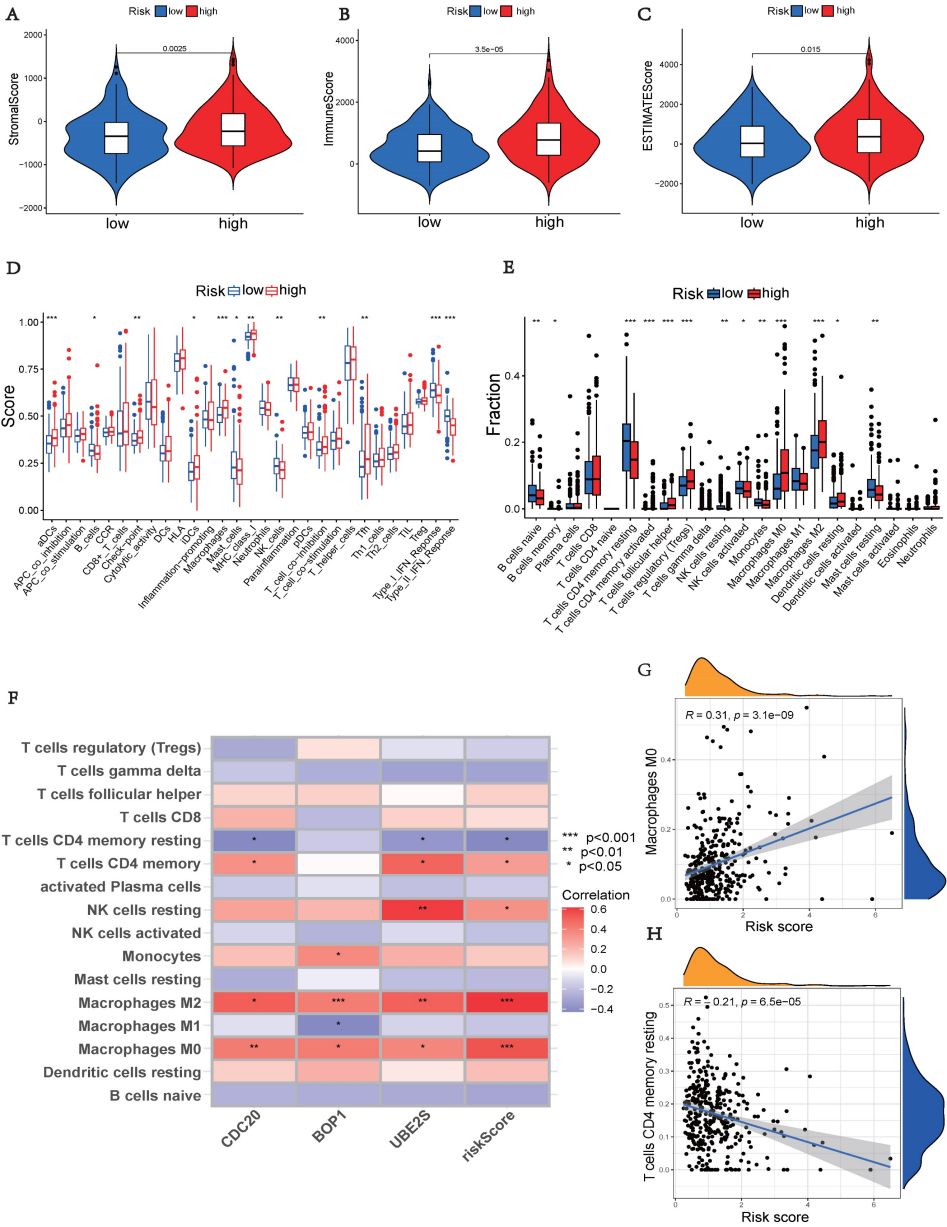
** Comparison of immune status between different risk groups.** (A) Comparison of stromal scores. (B) Comparison of immune scores. (C) Comparison of ESTIMATE scores. (D) Comparison of immune cell enrichment scores. (E) Comparison of immune cell infiltration. (F) Correlation analysis between immune cells and UbRGs. (G) Correlation analysis between risk score and macrophages M0. (H) Correlation analysis between risk score and T cells CD4 memory resting. (*p < 0.05; **p < 0.01; ***p < 0.001).

The high-risk group, showed significantly higher stromal, immunological, and ESTIMATE scores (*p* < 0.05), indicating an enhanced overall immune status and immunogenicity in the TME of the high-risk group (Figures 7A-C). To further explore the immune landscape across different risk groups, we employed CIBERSORT and ssGSEA for evaluation. The ssGSEA was used to quantify the enrichment scores of immune cell infiltration in different groups and examine the correlation between immune cells and their functions (Figure 7D). The CIBERSORT analysis revealed a significant enrichment of macrophages M0, CD8^+^ T cells, and macrophages M2 in the high-risk group, while activated B cells and resting CD4^+^ memory T cells were significantly enriched in the low-risk group (Figure 7E). Those results showed differences between the two risk groups in macrophages, T cell co-inhibition, type II IFN response, and other factors. Furthermore, we assessed the correlation between hub UbRGs and immune cells, as the abundance and functionality of immune cell infiltration significantly influence tumor immunotherapy. The majority of immune cells showed strong correlations with the three hub UbRGs and risk scores. Importantly, macrophages M0 and macrophages M2 exhibited the strongest and most significant correlation with the risk score (Figure 7F). The correlation scatter plots depicted the relationship between macrophages M0 and resting CD4^+^ memory T cells (Figures 7G-H).

The above findings indicate distinct immune landscapes between the two risk groups, and variations in immune response levels may reveal prognostic disparities in HCC.

### Immunotherapy Prediction

**Figure 8:**
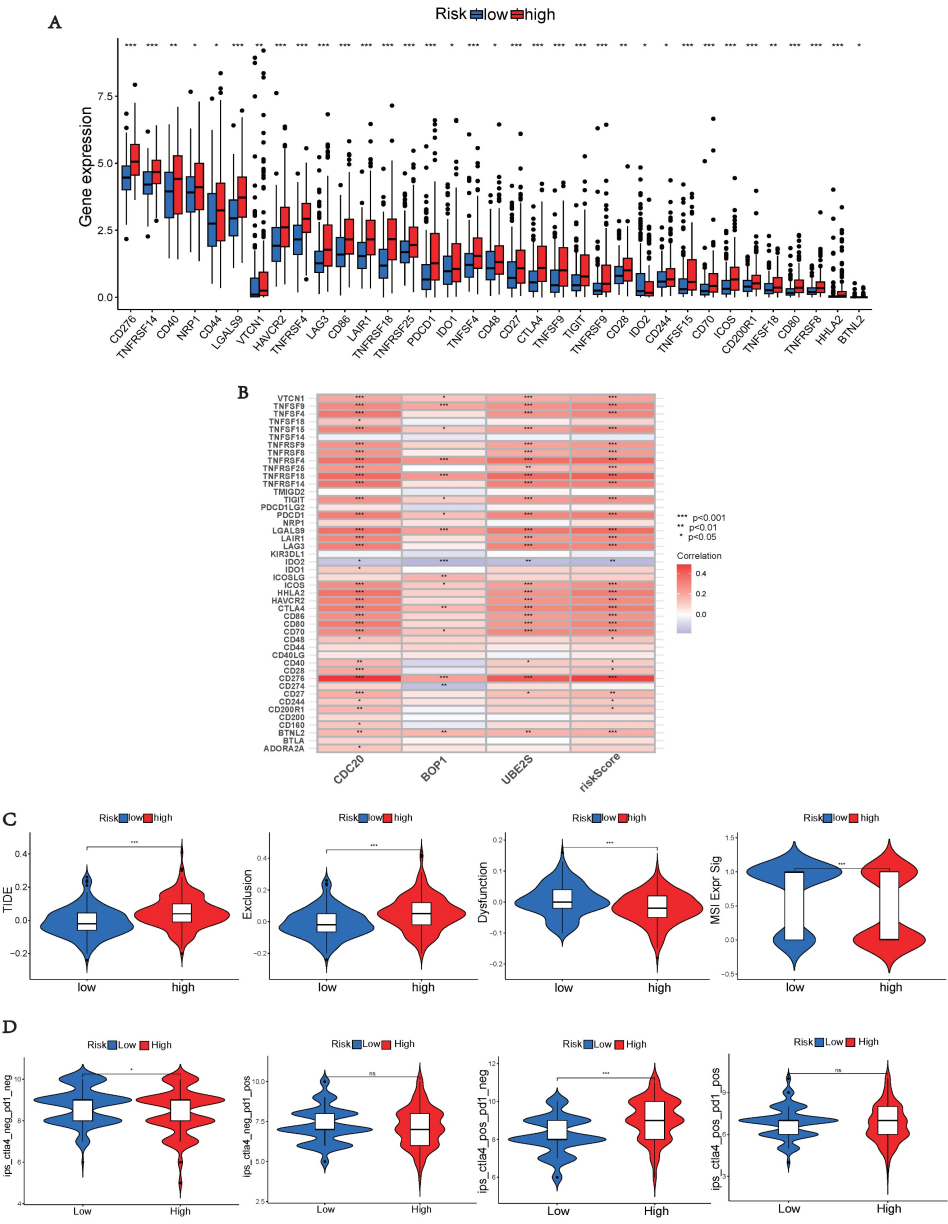
** Immune checkpoint analysis, TIDE and TCIA.** (A) Expression of immune checkpoints in different risk groups. (B) Association between immune checkpoints and UbRGs. (C) TIDE, scores for T cell exclusion and dysfunction, and MSI in different risk groups. (D) Differences in immune checkpoint inhibitor response between the high-risk and low-risk groups. (*p < 0.05; **p < 0.01; ***p < 0.001).

As immunological checkpoints play a critical role in tumor immunotherapy, we investigated the differences in immune checkpoint transcription between the two risk groups. High-risk patients demonstrated significant up-regulation of 33 immune checkpoint genes (Figure 8A), indicating their potential benefit from targeted therapies against up-regulated immune checkpoints. Subsequently, we explored the correlation between risk scores of hub UbRGs and positive immune checkpoints. Most immunological checkpoints and risk scores showed positive correlations, with CD276 having the strongest association, as indicated by the heat map (Figure 8B). We further conducted the TIDE analysis to investigate the interaction between TMB and immunotherapy and to determine potential differences in response to immunotherapy among patients with diverse risk patterns. The study results revealed that the high-risk group showed significantly elevated TIDE score and Exclusion score, as well as a lower MSI score (Figure 8C). These findings indicate an increased potential for immune escape in the high-risk group and suggest a potential poor efficacy of immune checkpoint inhibition therapy (ICI). Additionally, TCIA was used to predict the response to immunotherapy. Patients in the low-risk group with negative PD-1 and CTLA-4 expression responded better to immunotherapy than those in the high-risk group, whereas patients in the high-risk group with negative PD-1 expression and positive CTLA-4 expression responded more favorably to immunotherapy than those in the low-risk group (Figure 8D). These findings indicate that high-risk and low-risk patients show distinct immune statuses, and our hub UbRGs-based signature has the potential to identify individuals who are suitable for checkpoint inhibitor treatment.

### Drug Sensitivity Analyses

**Figure 9:**
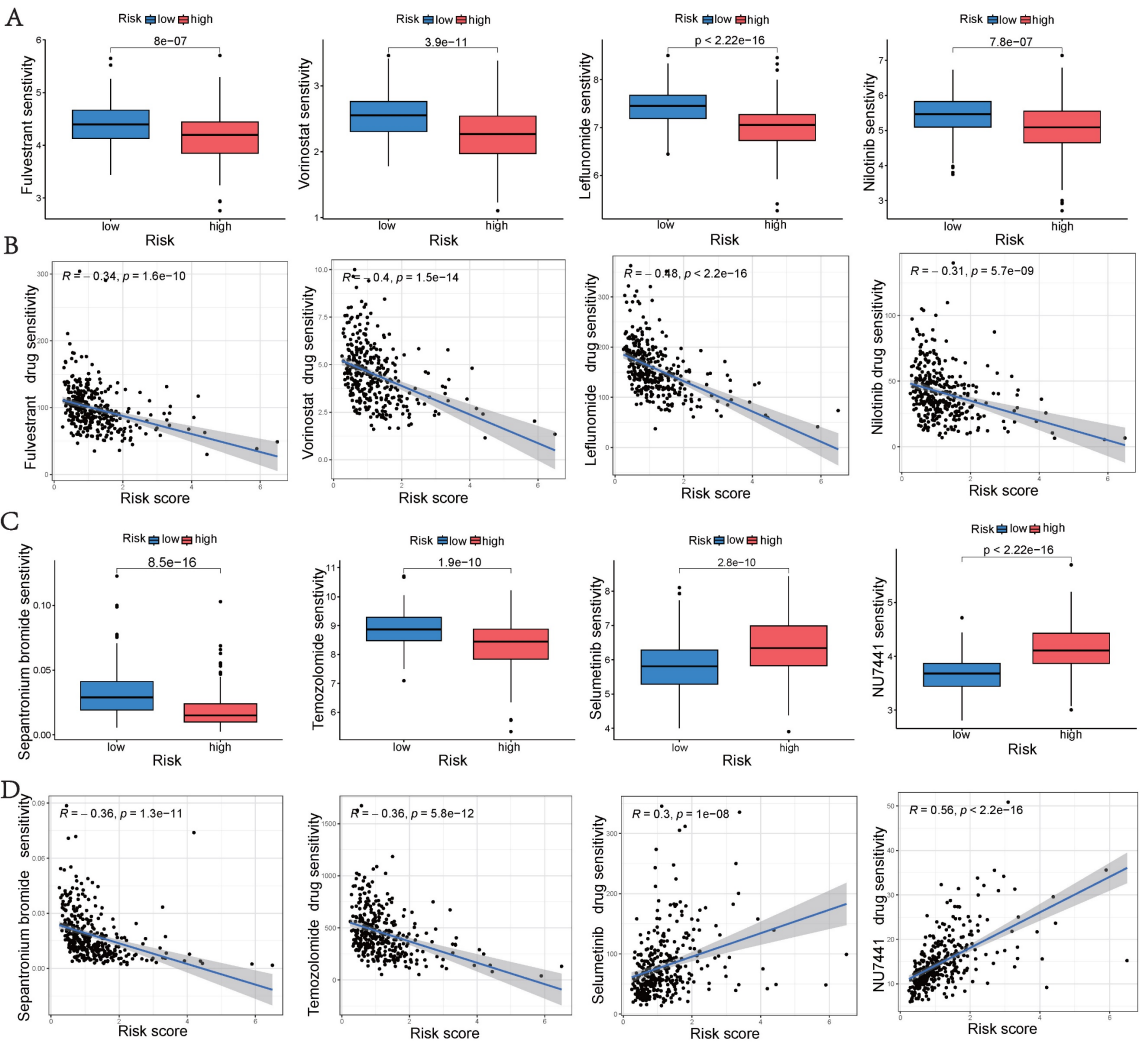
** Drug sensitivity analyse.** (A, C) Comparison of drug sensitivity between the low-risk and high-risk groups. (B, D) Correlation between risk scores and drug sensitivity. Statistical significance was defined as *p* < 0.05.

To aid in the development of personalized treatment programs, we evaluated the sensitivity of patients in different risk categories to selected chemotherapeutic drugs in the current clinical setting. High-risk patients exhibited lower IC50 values for fulvestrant, vorinostat, leflunomide, nilotinib, sepantronium bromide, and temozolomide compared to low-risk patients. This suggests that patients may potentially benefit more from the mentioned drugs in the high-risk group. Selumetinib and NU7441 exhibited higher IC50 values in the high-risk group, suggesting that these two chemotherapeutic agents were more resistant in the high-risk group (Figures 9A and 9C). Additionally, a significant correlation was observed between the risk score and drug sensitivity (Figures 9B and 9D). These findings emphasize the substantial potential of hub UbRGs in predicting chemotherapy sensitivity and indicate its potential value in guiding clinical treatment decisions.

### Single-cell Sequencing Data Analysis

**Figure 10:**
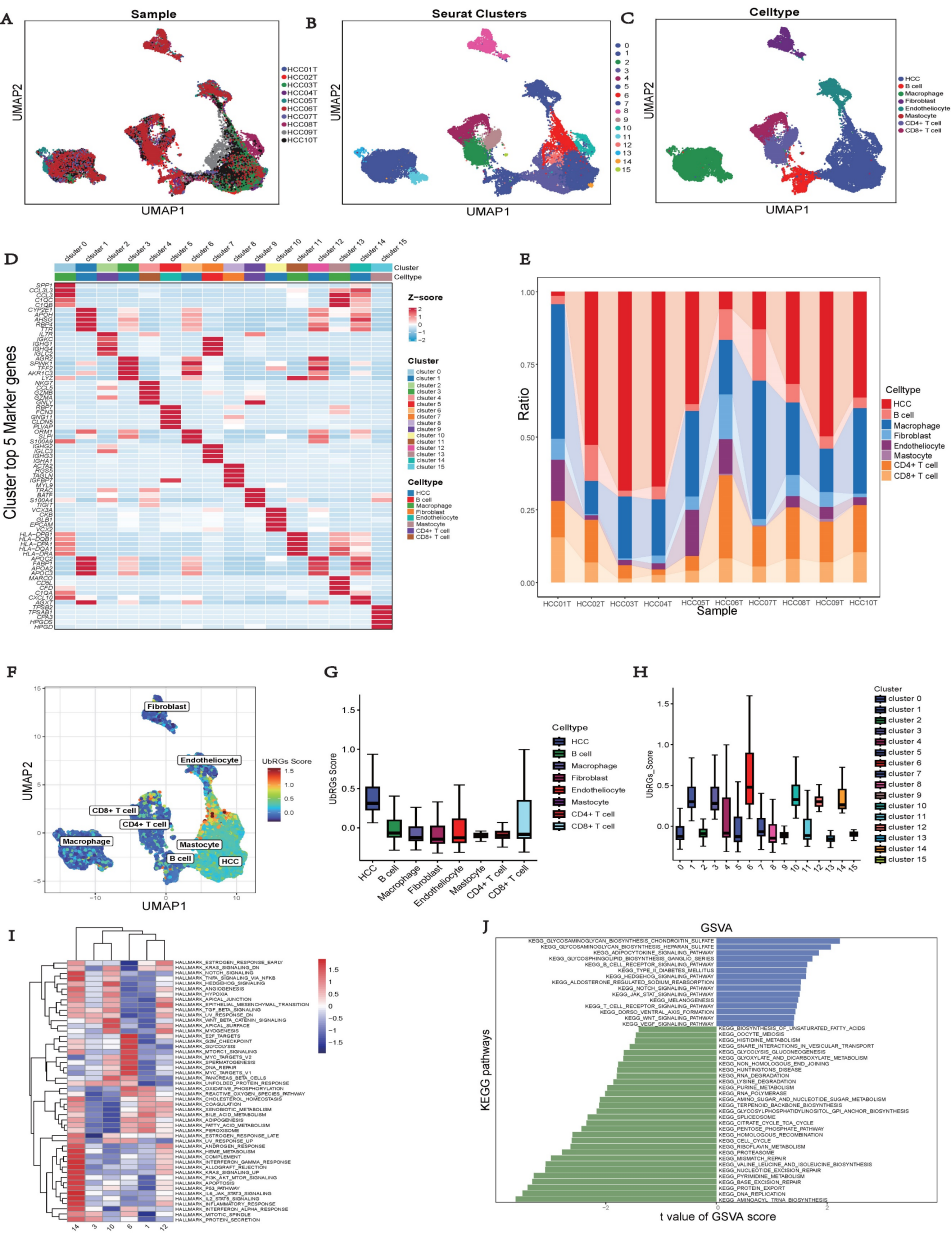
** Single-cell analysis of HCC sample.** (A) Different samples in the HCC dataset. (B) 34,061 cells belonging to sixteen cell clusters in ten HCC samples. (C) Different cell types. (D) Heatmap showing the expression of the top five marker genes for each cluster. (E) Frequency distribution of cell types across ten samples. (F) UMAP visualization of UbRGs expression in different cell subsets. (G) Boxplots illustrating the scores of UbRGs in different cell clusters. (H) Boxplots illustrating the scores of hub UbRGs in different cell clusters. (I) Heatmap showing different hallmark pathways enriched in HCC cell clusters by GSVA. (J) Differences in KEGG pathway activities scored per HCC cell by GSVA between hub UbRGs high and low score cells.

The GSE149614 dataset, comprising 10 HCC tumor samples, was downloaded from the GEO database to investigate the expression of hub UbRGs in tumor cells (Figure 10A). After performing quality control on each tumor sample, a total of 34,061 cells were included in the study. The quality-controlled cells were subsequently divided into 16 clusters (Figure 10B), and the differences among the cells within each cluster were analyzed. A heat map was generated to visualize the expression patterns of the top five most important genes (Figure 10D). Eight major cell types, including HCC, B cells, macrophages, fibroblasts, endothelial cells, mast cells, CD4^+^ T cells, and CD8^+^ T cells, were identified in the tumor samples (Figure 10C). Additionally, we provide the relative proportions of different cell types within the tumor samples. Upon examining the distribution of the eight cell lineages, the most predominant cell type in the tumor samples was HCC, followed by macrophages, CD4^+^ T cells, and CD8^+^ T cells (Figure 10E). Furthermore, UMAP analysis revealed heterogeneous levels of hub UbRGs enrichment in tumor cells and immune cells (Figure 10F), with the highest enrichment observed in HCC cells, followed by CD8^+^ T cells (Figure 10G). Cluster 6 exhibited significantly elevated UbRGs activity compared to other HCC cell subtypes (Figure 10H). Subsequent analysis revealed significant enrichments of the hallmark cell cycle (E2F targets, G2M checkpoint, MYC targets v2, DNA repair) and metabolism (glycolysis) pathways in cluster 6 (Figure 10I). Furthermore, DEGs between high and low UbRGs-scored HCC clusters exhibited significant enrichments in metabolism, angiogenesis, cell differentiation, and immune-related pathways, including glycosaminoglycan biosynthesis chondroitin sulfate, VEGF, WNT, B and T cell receptor signaling pathway (Figure 10J). Overall, these findings highlight the importance of hub UbRGs in the context of HCC cells and immune cells within TME.

### Validation of UbRGs Expression

**Figure 11:**
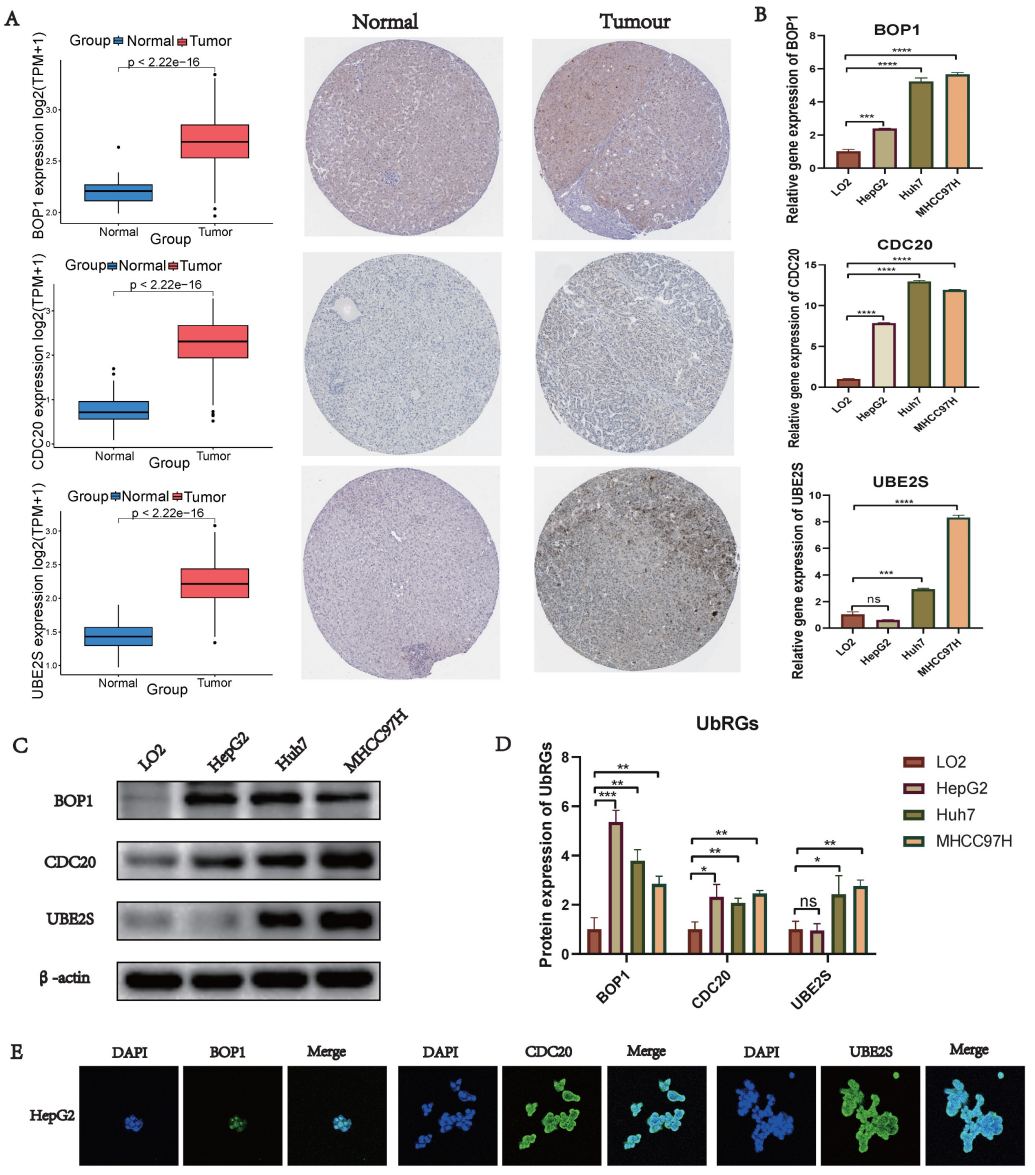
** Validation of UbRGs expression was based on HPA database and experiments.** (A) The expression profiles of UbRGs in normal and tumor tissues were retrieved from the TCGA database, and the immunohistochemistry images of UbRGs were obtained from the HPA database. (B) RT-qPCR was used to measure the expression levels of BOP1, CDC20, and UBE2S genes in the normal liver cell line (LO2) as well as in liver cancer cell lines (HepG2, Huh7, MHCC97H). (C) Western blotting (WB) was performed to detect the protein levels of BOP1, CDC20, and UBE2S in the normal liver cell line (LO2) as well as in liver cancer cell lines (HepG2, Huh7, MHCC97H). (D) The bar graph illustrates the expression differences of UbRGs among different cell types. (E) Immunofluorescence was used to determine the subcellular localization of UbRGs in tumor cells.

We conducted a combined analysis using the HPA database and cytological experiments to further validate the expression of hub genes for UbRGs. The HPA database was utilized to investigate the immunohistochemistry of BOP1, CDC20, and UBE2S in HCC tissue samples (Figure 11A). The results revealed higher staining intensity of CDC20 and UBE2S compared to normal liver tissue. Subsequently, RT-qPCR was used to evaluate the differences in gene expression levels between the normal liver cell line (LO2) and three liver cancer cell lines (HepG2, Huh7, MHCC97H). The results demonstrated the up-regulation of BOP1, CDC20, and UBE2S in liver cancer cells (Figure 11B). The western blot bands exhibited consistency with the RT-qPCR results (Figure 11C). The bar chart quantitatively illustrates the variation in protein levels of hub genes for UbRGs among different cell lines (Figure 11D). Considering the current studies, there is insufficient evidence regarding the cellular sublocalization of hub genes for UbRGs. Hence, we investigated HCC cells using IF. The results showed predominant expression of BOP1 in the nucleus, while CDC20 and UBE2S exhibited high expression in the cytoplasm (Figure 11E).

## Discussion

HCC accounts for 90% of liver cancer cases and has a 5-year survival rate of 18% 1, 20. Unfortunately, only 25% of HCC patients carry at least one potential targetable therapeutic mutation, while the majority of the primary cancer-causing genes remain untargetable 21. Furthermore, HCC does not typically respond well to conventional chemotherapies. As a results, hepatic resection and transplantation are the most effective treatments 22. New hepatic TME modulating therapies have emerged since 2017. Pembrolizumab and nivolumab, two PD-1-targeting immune checkpoint inhibitors, have received FDA approval as second-line therapies for advanced HCC 23, 24. However, despite these remarkable results, only 20%-30% of patients derive benefits from immunotherapies, and biomarkers have thus far failed to identify the responsive groups 25, 26. Identifying new therapeutic targets and prognostic biomarkers is an imperative necessity.

Ubiquitination is the covalent attachment of ubiquitin to lysine residues on target proteins, which serves as a post-translational protein modification 27. Mounting evidence suggests that ubiquitination, along with its reverse process, deubiquitination, plays crucial regulatory roles in innate and adaptive immune responses by modulating the functions of diverse immune cells. For instance, ubiquitination regulates apoptosis and autophagy in macrophages 28, MHC II expression in dendritic cells 29, TCR signaling and T cell activation 30, as well as B cell development and activation 31. Ubiquitination research has primarily focused on the phenotypic changes induced by individual proteins. However, studies investigating the multi-omics characteristics of UbRGs have been reported infrequently. To elucidate the biological function and clinical significance of ubiquitination in HCC, we conducted an analysis of the genomic and transcriptomic characteristics related to UbRGs.

We investigated the significance of UbRGs in HCC through bioinformatics analysis using RNA-seq and scRNA-seq techniques. Initially, we identified a signature of UbRGs using WGCNA and machine learning (Figure S2 and Figure 2). Three UbRGs (BOP1, CDC20, UBE2S) were identified using Cox regression, SVM-RFE, LASSO, and GBM algorithms. Subsequently, prognostic models were constructed based on these three genes (Figure 3). These ubiquitin hub genes are associated with the progression of HCC. BOP1, an RNA-binding protein, is involved in ribosome biogenesis, as well as cell cycle regulation and cell proliferation 32. Ubiquitination of hnRNPU mediated by CDC20 regulates chromatin condensation through the modulation of the CTCF-cohesin complex-hnRNPU interaction 33. Dysregulation of the CDC20-hnRNPU axis is implicated in tumor growth and treatment resistance 34. CDC20 has emerged as a promising therapeutic target for cancer treatment, as reported by researchers 35. UBE2S, a ubiquitin-conjugating enzyme, regulates cell cycle progression, apoptosis, and protein ubiquitination, which are directly associated with tumor growth 36. UBE2S serves as a predictive biomarker for patients, and the UBE2S-PTEN-pAKT signaling axis holds promise as a therapeutic target for HCC 37. Nevertheless, there are no published studies investigating the association between these three potential ubiquitin hub genes and prognosis in HCC patients, specifically for the construction of prognostic models. We believe that our study can offer valuable insights for future clinical decision-making processes.

Prognostic models for HCC were developed using the signature of UbRGs (BOP1, CDC20, and UBE2S). The model categorizes patients into high and low risk subgroups, which exhibit significantly different prognoses (Figure 3A). This classification was achieved by evaluating each patient's risk score based on the median risk values. ROC curves were generated using different datasets, and the AUC values were calculated at 1, 3, and 5 years. Among these, the highest AUC value of 0.845 was observed (Figure 3E). Multivariate analysis indicated that the new signature could potentially serve as an independent prognostic factor (Figure 4A). For precise and efficient quantification of HCC patients' clinical characteristics, we developed a nomogram incorporating age, gender, stage, and risk score. This nomogram, illustrated in Figure 4B, accurately predicts patients' overall survival and facilitates more precise risk assessment, aiding in diagnosis and treatment decisions. The calibration curves of the established nomogram demonstrated a high level of accuracy in aligning actual observations with predictions (Figure 4C). Furthermore, clinically relevant ROC curves provide evidence that risk scores outperform other clinical features in terms of their efficacy for clinical applications (Figures 4D-F). Consistent with conventional clinical staging, the high-risk group had a higher proportion of patients in stage II-III. These findings suggest that the model can provide improved predictions of patient prognoses.

Immune interactions are crucial features of tumorigenesis and represent a therapeutic target for HCC. Immune cells and stromal cells are the major constituents of TME, and both immune scores and stromal scores have been found to correlate with clinicopathological characteristics and prognosis 3. Pathway analysis showed that the high-risk group identified by the ubiquitination score was enriched in pathways related to the immune system (Figure 6). By utilizing the ESTIMATE algorithm, we calculated these scores and found that the high-risk group had significantly higher immune and stromal scores (Figures 7A-B). These results suggest a potential association between ubiquitination and the TME, which may influence the initiation and progression of neoplasia. The relative abundance of 22 immune cell groups in the HCC samples was determined using the CIBERSORT method. The activation of immune cells can impact prognosis. The high-risk group exhibited higher rates of immune cell infiltration compared to the low-risk group, specifically showing increased abundance of macrophages M0, macrophages M2, and T cells CD8. Macrophages, T cell co-inhibition, and type II IFN response exhibited differences among risk groups as determined by ssGSEA (Figure 7D). The correlation analysis between immune cells, expression of hub UbRGs, and risk scores revealed a strong positive correlation between macrophages and ubiquitination genes (Figure 7F). M2 macrophages are recognized as crucial contributors to tumor progression and have been associated with poor prognoses 38. Ubiquitin promotes macrophage M2 polarization and activates the CXCR4/ERK signaling pathway, thereby facilitating the invasion of HCC 39. Serpinc1 suppresses HCC by inducing apoptosis and inhibiting macrophage polarization through the ubiquitin-proteasome system 40. These findings suggest that the ubiquitination process in M2 macrophages plays a critical role in HCC.

Cancer cells employ checkpoints to suppress T cell reactivity and evade immune destruction 41. The success of treatment with immune checkpoint inhibitors can be predicted by assessing TMB, which serves as an indicator of the mutational burden in cancer 42, 43. The major histocompatibility complex proteins present neoantigens to T cells following mutations. In this study, we observed that the high-risk group with a high TMB exhibited the poorest prognosis (Figure 5E). Immune checkpoints are critical for immune responses 44. We observed a significant up-regulation of immunological checkpoint-related genes in the high-risk group (Figure 8A). The high-risk group exhibited a higher TIDE score, suggesting a greater potential for immune escape and less effective immune checkpoint inhibition in these individuals (Figure 8C). Furthermore, patients from the IMvigor210 cohort who responded to anti-CTLA-4 treatment showed a significant decrease in UbRGs (Figure 8D). Responses to immunotherapy, as indicated by PD-1 and CTLA-4 expression, differed across risk groups. Signatures based on hub UbRGs may identify patients who could benefit from checkpoint inhibitor therapy, thus aiding in the advancement of targeted liver cancer treatments and enhancing immunotherapy approaches. Finally, we evaluated the drug sensitivity of each potential regulator (Figure 9). Chemotherapy sensitivity varied among patients in different risk groups, with notable correlations between risk scores and drug response (Figures 9B and 9D). Utilizing UbRGs, we can foresee chemotherapy response in high and low-risk groups, aiding clinicians in devising personalized treatment strategies. Precise forecasts in drug discovery and development can optimize time and resource utilization 45. Ubiquitination hub genes can predict the immunotherapy and medication sensitivity responses of patients.

This research had several limitations. Firstly, we confirmed the expression of hub UbRGs in HCC cells through cytological experiments at both the gene and protein levels. To elucidate the specific underlying mechanisms, further in vitro experiments need to be conducted. Secondly, despite constructing a prognostic signature and validating it using both external and internal datasets, the possibility of source bias remains. Therefore, additional clinical cohorts are required to validate our findings. Thirdly, our model is based on three specific UbRGs and the roles of additional UbRGs in HCC biology warrant further investigation. Nonetheless, this study can still provide valuable assistance to clinicians in risk stratification of patients and in selecting appropriate therapies.

## Conclusion

In conclusion, our findings suggest that the model incorporating the UbRGs signature can accurately predict the prognosis of patients with HCC. Furthermore, we have confirmed the expression of UbRGs signature in HCC through cellular experiments. The UbRGs signature shows promise as a novel biomarker, with potential applications in prognosis assessment, immunotherapy, and drug sensitivity prediction for HCC patients. These findings contribute to the understanding and potential clinical implications of the UbRGs signature in HCC management. Further studies are warranted to validate and explore the full potential of the UbRGs signature in improving patient outcomes.

## Supplementary Material

Supplementary figures.

Supplementary tables.
