# Supplementary material for: Prognostic and Immune Landscape Analysis of Ubiquitination-related Genes in Hepatocellular Carcinoma: Based on Bulk and Single-cell RNA Sequencing Data
Source: J Cancer. 2024 Mar 11;15(9):2580–600. doi: 10.7150/jca.93425 (PMC10988309; doi:10.7150/jca.93425)
Supplement: Supplementary file 1 — Supplementary figures. [file jcav15p2580s1.pdf]

Supplementary Figures

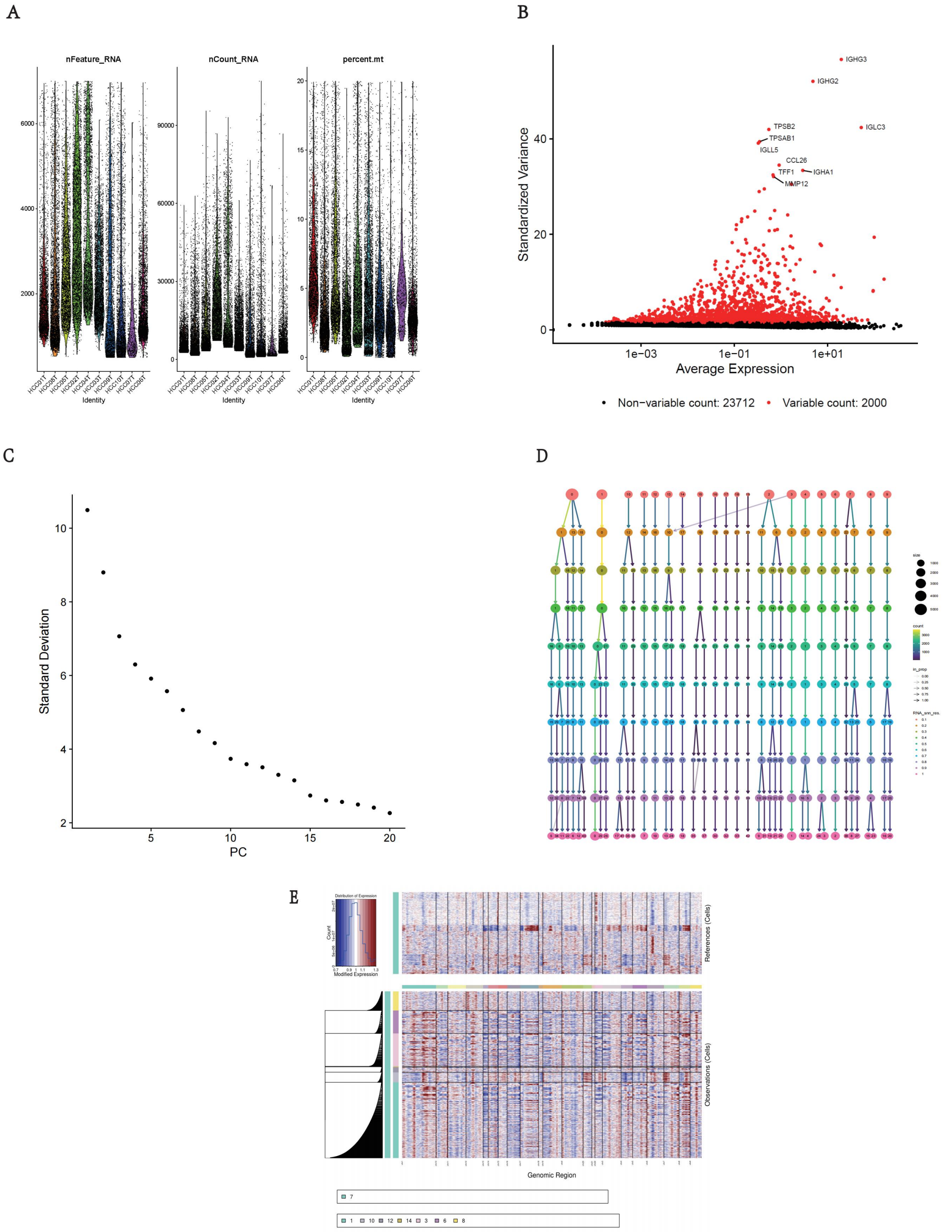

Figure S1. scRNA-seq data processing. (A) Quality control of GSE149614 data set. (B) Identification of highly variable features. (C) Canking of principle components based on the percentage of variance explained. (D)

Cluster tree of 34061 cells from scRNA-seq. (E) Heatmap showing CNV profile of clusters. Red and blue colors represent high and low CNV level, respectively.

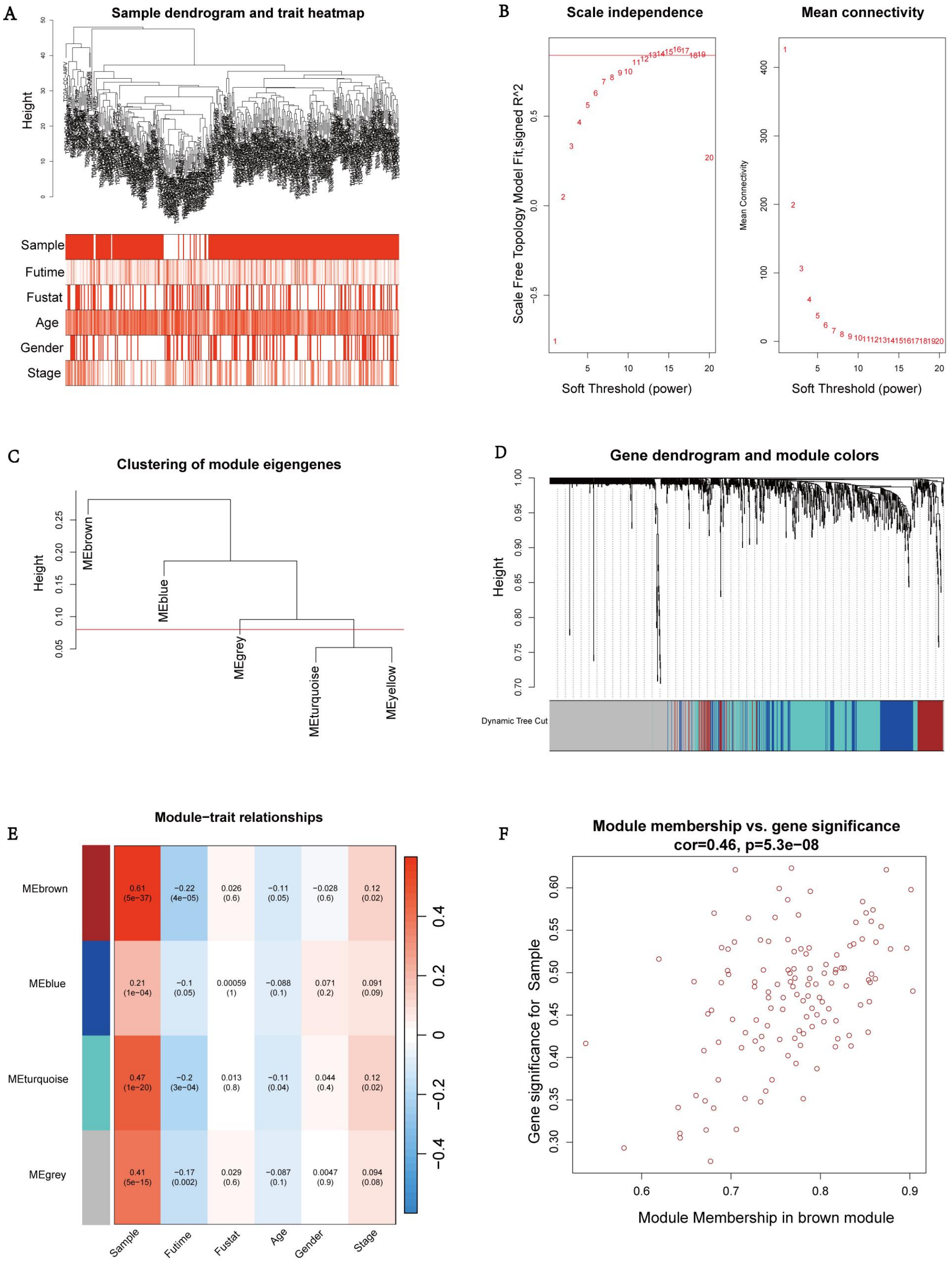

Figure S2. Analysis of gene co-expression using weighted network analysis. (A) Dendrogram and sample trait indicators based on Pearson correlation coefficients calculated between samples. (B) Screening for appropriate soft thresholds and validation of a scale-free network. (C) Hierarchical clustering analysis of gene modules using WGCNA. (D) The dendrogram illustrating the clustering of gene modules and their merging. (E) The relationship between gene modules and clinical features. (F) The correlation between gene significance (GS) and module membership (MM) is visualized as a scatter plot in the MEblue modules.
